# Aurum in Sarcocele

**Published:** 1895-08

**Authors:** 


Aurum in Sarcocele.—A man had a hard enlarged testicle on the right side, painful to touch. Aurum-met., 15th trit., was given three times a day; the testicle gradually took its normal size and became softer, and in six weeks patient was discharged *cured.—North American Journal of Homœopathy*, April, p. 249.

